# Metatranscriptomic Approach to Analyze the Functional Human Gut Microbiota

**DOI:** 10.1371/journal.pone.0017447

**Published:** 2011-03-08

**Authors:** María José Gosalbes, Ana Durbán, Miguel Pignatelli, Juan José Abellan, Nuria Jiménez-Hernández, Ana Elena Pérez-Cobas, Amparo Latorre, Andrés Moya

**Affiliations:** 1 Unidad Mixta de Investigación en Genómica y Salud-Centro Superior Investigación en Salud Pública (Generalitat Valenciana)/Instituto Cavanilles de Biodiversidad y Biología Evolutiva (Universitat de València), València, Spain; 2 CIBER en Epidemiología y Salud Pública, València, Spain; Institut Pasteur, France

## Abstract

The human gut is the natural habitat for a large and dynamic bacterial community that has a great relevance for health. Metagenomics is increasing our knowledge of gene content as well as of functional and genetic variability in this microbiome. However, little is known about the active bacteria and their function(s) in the gastrointestinal tract. We performed a metatranscriptomic study on ten healthy volunteers to elucidate the active members of the gut microbiome and their functionality under conditions of health. First, the microbial cDNAs obtained from each sample were sequenced using 454 technology. The analysis of 16S transcripts showed the phylogenetic structure of the active microbial community. *Lachnospiraceae*, *Ruminococcaceae*, *Bacteroidaceae*, *Prevotellaceae*, and *Rickenellaceae* were the predominant families detected in the active microbiota. The characterization of mRNAs revealed a uniform functional pattern in healthy individuals. The main functional roles of the gut microbiota were carbohydrate metabolism, energy production and synthesis of cellular components. In contrast, housekeeping activities such as amino acid and lipid metabolism were underrepresented in the metatranscriptome. Our results provide new insights into the functionality of the complex gut microbiota in healthy individuals. In this RNA-based survey, we also detected small RNAs, which are important regulatory elements in prokaryotic physiology and pathogenicity.

## Introduction

The gastro-intestinal (GI) tract is an essential metabolic organ that is populated with a huge number of microbes. The intestinal microbiota is important for human health because of nutrient processing, development of the immune system, colonization resistance and stimulation of a variety of other host activities [1], [2], [3], [4].

Our knowledge about bacterial diversity in the human GI tract has increased concomitantly with the development of different molecular approaches such as fingerprinting techniques of 16S rDNA amplicons, sequencing of 16S rDNA clones, fluorescent in situ hybridization, DNA microarrays or, more recently, high-throughput sequencing [5], [6], [7], [8], [9], [10], [11], [12], [13], [14], [15], [16], [17]. All these studies have shown that the composition of the intestinal microbiota varies between individuals due to host genotype, age, health status and diet, though the predominant population is fairly stable under normal conditions. We also know that the predominant bacterial groups in the human GI tract are *Bacteroidetes*, *Firmicutes* and *Actinobacteria*, and that substantial variability exists in the particular bacterial lineages carried by an individual [9], [10], [12], [14], [16], [17], [18]. Since the GI microbiota is highly diverse and variable across individuals, it is difficult to establish the relationship between particular microorganisms and health status. The stability of the GI microbiome is a function, not only of its composition, but also of the gene expression of its members. It is therefore essential to explore the gene expression of the microorganisms in the GI tract.

Recently, metagenomics applied in a variety of microbial habitats, including the GI tract, have led to the discovery and characterization of new genes from uncultivated microorganisms, assembly of whole genomes from community DNA sequence data and comparison of microbial community composition between different environments [9], [14], [17], [19], [20], [21], [22], [23], [24], [25], [26], [27], [28]. Although metagenomic data provide extensive information about microbiota diversity, gene content and their potential functions, there is no indication on whether DNA comes from viable cells or whether the predicted genes are expressed at all and, if so, under what conditions and to what extent.

Environmental metatranscriptomics retrieves and sequences environmental mRNAs from a microbial ecosystem to assess what genes may be expressed in that community. To date, metatranscriptomic studies have been applied mainly to samples from water and soil environments [29], [30], [31], [32], [33], [34], [35]. In the GI ecosystem, the diversity of gut microbiota has been the subject of many metagenomics studies but only a few have focused on the active microbiota in the human gut. cDNA microarrays have been used in different systems to explore bacterial activity from particular species in the intestinal tract. Mahowald *et al.* [36] performed whole-genome transcriptional analysis of colonic RNA prepared from mice that were germ-free or colonized with *Bacteroides thetaiotaomicron* (*Bacteroidetes*) and *Eubacterium rectale* (*Firmicutes*) using the bacterial Genechip. Klaassens *et al.* [37] applied a *Bifidobacterium*-specific microarray to infant feces revealing that bifidobacterial species undergo differential transcriptional responses depending on the diet. Recently, metatranscriptomic analysis has been applied to two fecal samples of a monozygotic twin pair [38]. In other study, the technique cDNA amplified fragment length polymorphism (cDNA-AFLP) was applied to a gene expression analysis of two healthy individuals [39].

The expression of prokaryotic genes remains difficult to study mainly due to problems related to the isolation of mRNA [40], [41], [42], [43], [44]. The half-life of mRNA is short and it is usually a small fraction of the total RNA. In addition, mRNA enrichment is challenging in prokaryotes, as prokaryotic mRNA lacks the 3′-end poly (A) tail that marks mature mRNA in eukaryotes. Furthermore, it is important to take into account that metabolically active bacteria contain more ribosomal RNA than latent or starved cells [45]. Because of this fact analyzing the ribosomal RNA transcripts of an ecosystem identifies the active members of the microbiota and provides a general picture of their differential activity levels.

Here, we report the metatranscriptomic study of the human GI tract microbiota in ten healthy individuals to elucidate a functional profile. We applied large scale pyrosequencing of the RNA community and used 16S rRNA transcripts as a marker of the phylogenetic structure of the active bacterial community. We also analyzed the protein-coding fraction to characterize the functions present in this habitat and the microorganisms involved in them. Additionally, this RNA-based approach allowed us to find, for the first time, untranslated regulatory elements in the gut microbial community.

## Results and Discussion

### Sequence identification

To study the functional fraction of the bacterial community we purified the total RNA of 10 fecal samples from healthy volunteers (Table S1 in SI). Although faeces may not present the same growing conditions (nutrient availability or oxygen concentration) as the gut mucosa, they may probably recover a substantial proportion of the bacterial species living in this environment and their activity. Moreover, it is easy to collect human fecal samples compared with the alternative invasive procedures to sample the contents of the gut. For these reasons we opted for the use of fecal samples as in almost all metagenomic and metatranscriptomic gut studies [9], [10], [14], [15], [16], [17], [38].

The cDNAs prepared from amplified mRNA ranged in size from 100 bp to 1 kb, the majority being between 200 and 500 bp. The pyrosequencing of all the samples yielded approximately 8,530,000 bp from 489,307 reads (174 bp average length) (Table 1). We only considered those sequences with high quality parameters. Additionally, we filtered out the reads that were shorter than 60 nucleotides, retaining a total of 409,503 reads. We set up a step-wise analysis to detect the different RNA types, such as rRNAs, mRNAs and other non coding RNAs, in order to study them separately. Firstly, these reads were compared against the Small Subunit rRNA Reference Database (SSUrdb) described by Urich *et al.* [34]. Secondly, all sequences that remained unassigned as SSU rRNA were analyzed with the Large Subunit rRNA Reference Database (LSUrdb) [34]. We obtained that 17.23% of the total number of sequences corresponded to 16S cDNAs from active bacteria and 0.47% to eukaryote 18S rRNA. However, the number of sequences assigned to LSU was between two- and five-fold the number of reads corresponding to 16S cDNA (Table 1). The partial fragmentation in the purification step could partly explain the percentage of rRNAs recovered, especially high in the case of LSU. Then, the non rRNAs represented 6.8% of the total cDNAs. Although the methodology employed allows enrichment in non-ribosomal RNAs, it is very difficult to completely remove rRNAs. A recent metatranscriptomic study of two fecal samples obtained similar results for rRNA depletion with the subtractive hybridization method, mapping only 5% of the cDNA reads to a coding sequence [38]. As mentioned earlier, many challenges are associated with RNA extraction. These arise in part from sample collection and processing, but also from characteristics of prokaryotic mRNAs. Some of these issues can be improved but others are inherent to the specific community sampled.

**Table 1:** Pyrosequenced cDNAs analyzed in this study.

	Sample A	Sample B	Sample C	Sample D	Sample E	Sample F	Sample K	Sample L	Sample N	Sample O
**Total number of reads**	57,300	48,150	34,849	19,625	17,891	22,748	69,100	75,059	35,276	29,505
**Total base pairs, Mb**	10.7	8.4	6.6	5.1	4.3	4.6	15.3	16.4	7.5	6.4
**SSU rRNA reads**	11,528	4,524	7,726	6,663	5,585	3,221	12,707	8,024	6,482	6,057
**LSU rRNA reads**	43,622	41,888	25,359	11,896	10,948	16,494	52,981	59,935	24,587	21,353
**Non rRNA reads**	2,150	1,738	1,764	1,066	1,358	3,033	3,412	7,100	4,207	2,095

Only sequences longer than 60 bp were considered.

### Taxonomic assignment of 16S rRNA transcripts

**Figure 1:**
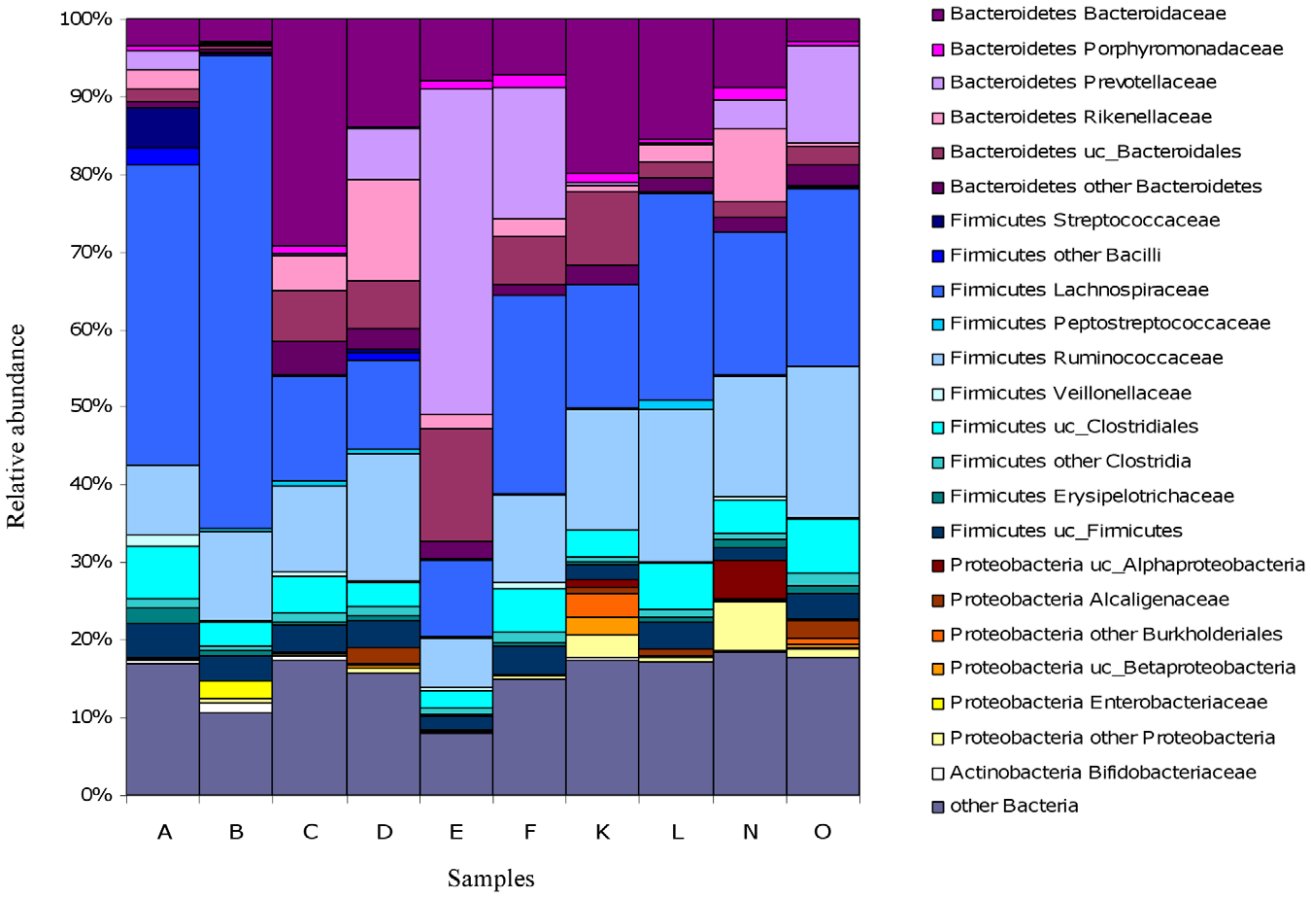
Composition of active microbiota. The composition for each sample is based on the taxonomic assignment of 16S transcripts.

To study the taxonomic classification of the active microbiota in fecal samples, each read previously assigned as a 16S transcript (70,593 sequences) was classified with The Ribosomal Database Project-II (RDP) [46]. Looking at the relative abundance of the rRNA sequences, we observed that the archaeal community, with *Methanobacteriaceae* as the only family found, was poorly represented in our samples in concordance with other molecular analyses [7], [10], [12], [47]. We also found that the two bacterial phyla, *Firmicutes* (49.18%) and *Bacteroidetes* (31.42%), provided the largest number of 16S rRNA transcripts in the functional communities analyzed. *Proteobacteria* (3.66%), *Actinobacteria* (0.4%) and *Lentisphaerae* (0.22%) were the other active phyla detected, though they accounted for fewer sequences (Figure 1). The low abundance of microaerobic proteobacterial sequences is consistent with the strict anaerobic environment of the colon. Interestingly, although the phylum *Actinobacteria*, mainly represented by *Bifidobacteria*, has been reported to be involved in protection against pathogens, maintenance of immune system and the exertion of nutritional effects for the intestinal cells and the host [2], [3], [47], [48], [49], we barely identified cDNA sequences of members of this phylum in our samples. This result could be due to a very low abundance or even absence of active *Bifidobacteriaceae* family as reported in a previous study [38].

Most of the *Firmicutes* sequences belonged to the order *Clostridiales*, being *Lachnospiraceae* (23.56%) and *Ruminococcaceae* (13.61%) the most represented families. These two families are known as pectin and cellulose degraders important in colonic fermentation of dietary fibers [50]. *Bacteroidaceae* (12.26%), *Prevotellaceae* (6.53%) and *Rickenellaceae* (3.61%) families, belonging to the phylum *Bacteroidetes*, appeared as the other functionally significant components of the human fecal microbiota (Figure 1). The *Prevotellaceae* family contains carbohydrate-fermenting and H_2_-producing bacteria implicated in energy production [50], [51]. Figure S1 shows the distribution of the main active families in each sample and the average value calculated from the global community composition. It can be seen that the most active families were the same in all samples, despite a certain level of between-sample variability.

### Richness and diversity in the active microbiota

To ascertain whether all the active families present in the samples had been recovered, a rarefaction analysis was carried out (Figure S2 in SI). The rarefaction curves show the rate at which new families are observed as sequencing continues. The curves suggest that we have observed most of the functional families present in all the samples except for samples O and L, where a few seem to be missing. Richness was estimated by two different estimators, the Chao1 and the abundance-based coverage estimation (ACE) (Table S2 in SI) [52], [53]. The comparison of the observed and estimated number of families also indicates, in agreement with the rarefaction curves, that we have observed most of the families present in all samples except for samples O and L. The Shannon index of biodiversity (H), which correlates positively with family richness and evenness, was also calculated at family level (Table S2 in SI) [54]. This estimator ranges between 1.9 and 2.4 for all the samples except for sample B where it is lower (1.5), indicating that in this sample there are fewer families and that they are more heterogeneously distributed than in the other samples. We applied correspondence analysis to explore patterns of variation in the family distribution between samples. Figure S3 shows that the samples were relatively homogeneous in bacterial composition. Overall, the functional microbiota is represented by a low number of bacterial families that are similarly distributed across samples.

### Functional analysis of putative mRNAs

We applied a transcriptomic approach to assess the potential functions of the RNA sequences found in our samples. The 27,923 cDNAs of the 10 samples that did not give a significant hit against the rRNA databases (SSUrdb and LSUrdb) were compared to the National Center for Biotechnology Information non-redundant protein database (NCBI-nr) using BLASTX [55]. Homologues to 14,680 sequences were found. The taxonomic assignments of putative mRNAs were predicted using the MEGAN software at the family level [56]. *Ruminococcaceae*, *Lachnospiraceae* and *Clostridiaceae* (*Firmicutes* phylum) together with *Bacteroidaceae*, *Rikenellaceae*, *Porphyromonadaceae* and *Prevotellaceae* (*Bacteroidetes* phylum) represented the most active families (Figure S4 in SI). The families *Bacteroidaceae* (36.17%), *Porphyromonadaceae* (2.53%), *Clostridiaceae* (1.87%) and *Bifidobacteriaceae* (0.56%), showed a higher relative abundance than in the 16S transcript analysis based on RDP. Conversely, the *Lachnospiraceae* family presented a drastic reduction in its relative abundance compared to the RDP analysis. We also analyzed the 16S transcripts with MEGAN, revealing differences between RDP and MEGAN assignments at family level (Figure S4 in SI). These discrepancies, described also by Claesson *et al.* [57], could be due to the distinct databases used, the differences between the Bergey (RDP) and NCBI (MEGAN) taxonomies and differences between the BLAST plus LCA (MEGAN) and Bayesian (RDP) algorithms. In spite of this variation in the relative abundances, the same families appeared as active bacterial members of the gut microbiota in both assignment methods.

Several studies reported that viruses represent an important constituent of human feces [58], [59]. In three samples (A, L, O) we found sequences with a viral assignment to the *Virgaviridae* family. However, this plant ssRNA virus family, diet-related, represented only a small fraction (0.1%–0.3%) of their respective assigned sequences. The characterization of the gut viriome warrants its own study.

**Figure 2:**
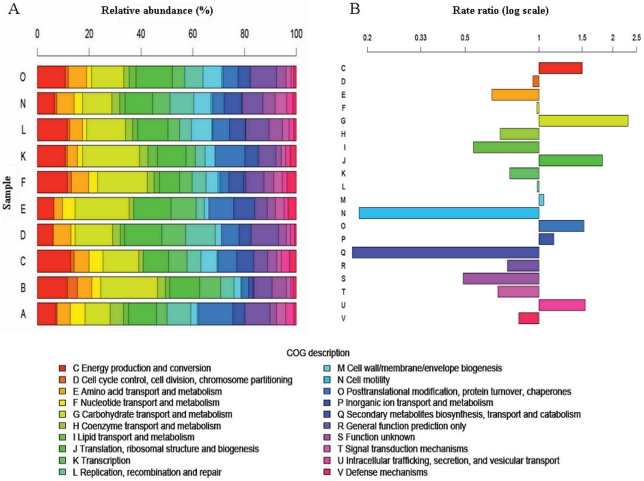
Analysis of COG assignment of mRNAs. (A) Distribution of COG categories across each sampled metatranscriptome. (B) Rate ratios of COG categories in the overall metatranscriptome. Rate ratios were calculated using (n_c_/n)/(N_c_/N), where n_c_ is the number of hits to a given category “c” in our samples, n is the total number of hits in all categories in our samples, N_c_ is the number of hits to that category in gCOGdb and N is the number of hits to all categories in gCOGdb.

To explore the potential function of the gut microbiota in the 10 fecal samples we analyzed the microbial metatranscriptomes obtained. The non ribosomal transcripts from each sample were searched by BLASTX against the gCOGdb obtained from all the completely sequenced bacterial genomes at NCBI and then including those of gut microbiota (see Materials and Methods). A total of 6,975 sequences (47.5%) were assigned to COG categories. This value is similar to the percentage of COG-assigned genes (51%) obtained in a cDNA-AFLP analysis of two fecal samples [39]. In other metatranscriptomic analysis of two fecal samples, it has been reported a high number of coding sequences unassigned to COG categories [38]. As well, the percentages of COG-assigned genes from three different metagenome studies ranged from 48% to 54% [9], [14], [17]. Figure 2A showed the functional distribution for each sample. In all the samples, the functional COG categories better represented were those corresponding to the functions: carbohydrate transport and metabolism, translation, ribosomal structure and biogenesis and energy production and conversion. However, other categories, such as lipid transport and metabolism, cell motility, secondary metabolite biosynthesis, transport and catabolism were poorly represented or even missing in some samples. Booijink et al. recently showed, using cDNA-AFLP analysis, that most of the annotated transcripts of two fecal samples were included in carbohydrate metabolism [39]. Our results indicated that the main functional roles of the gut microbiota in the 10 healthy individuals studied are related to nutrient processing, energy production and synthesis of cellular components, as suggested in previous DNA-based metagenomic analyses [16], [17] and a proteome-level study [60]. To assess whether the COG distribution found for each sample was an artifact of the reference database content we analyzed the over- or under-representation of COG categories in the pooled metatranscriptome with respect to that database (see Materials and Methods) (Figure 2B). We observed an over-representation of COGs classified into the carbohydrate transport and metabolism category (G) and an under-representation of COGs for the lipid transport and metabolism group (I). This has been reported previously in different surveys of the gut microbiota [9], [14], [16], [17], [38], [39], [60]. This profile indicates that the principal source for energy production and biosynthesis of cellular components in the microbiota comes from the fermentation of polysaccharides or dietary fiber, which results in the production of short-chain fatty acids that are then used by the host as an energy source. However, we did not find an over-representation of the amino acid transport and metabolism category (E) as described in previous metagenomic studies. Additionally, we found that the inorganic ion transport and metabolism functional category (P) is over-represented in our survey in contrast to the metagenomic data [9], [16], [17]. These discrepancies could be due to real differences among the different individuals included in each study, or to the nature of the molecules analyzed, DNA in metagenomics and mRNA in this study.

**Figure 3:**
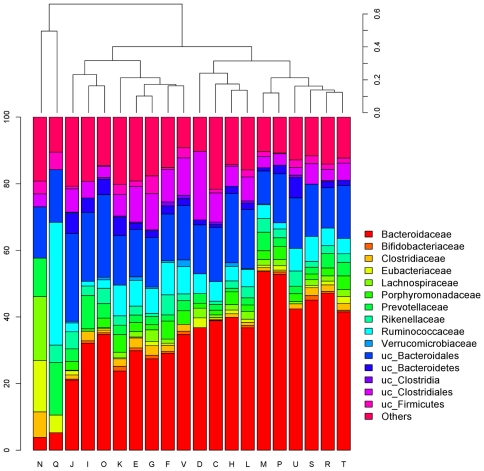
Distribution of taxa in functional groups. Barplot (bottom) and hierarchical cluster grouping functions according to the estimated taxa distribution profiles (top).

The functional contribution of the bacterial families in each COG category is shown in Figure 3. *Bacteroidaceae* appeared as the main family involved in nearly all the functional categories. Moreover, the distribution of the families along the categories is rather similar. Cell motility (N) and Secondary metabolite biosynthesis, transport and catabolism (Q) categories presented the most uneven family distribution. Cell motility category is generally under-represented (Figure 2B), as motility is not required by intestinal bacteria to persist in the gut due to the constant peristaltic movements, and the only sequences found in this category were assigned to families described as flagella producers. On the other hand, *Ruminococcaceae* and *Prevotellaceae* were the major families involved in the secondary metabolite biosynthesis, transport and catabolism category. The homology search by BLASTX against nr-NCBI database of the sequences assigned to these two families revealed that the *Ruminococcaceae* family was mainly associated with antibiotic biosynthesis while *Prevotellaceae* was related to the transport of secondary metabolites. These two findings together might reflect the importance of the gut microbiota in the defense against pathogens and in the maintenance of a healthy status.

**Figure 4:**
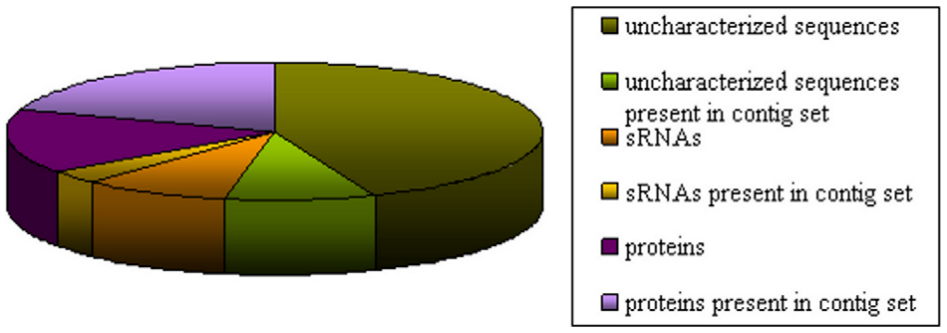
Homology search of unassigned transcripts.

Recently, Qin et al. [17] described deeply the gut metagenome from fecal samples of 124 European individuals. They applied Illumina-based sequencing and obtained 567.7 Gb of sequences and 3.3 million open reading frames, generating an extensive catalogue of sequences. To assess the representativeness of the sequences that are not rRNAs in the contig set described by Qin et al. [17], we searched homology by BLAT [61] between them (Figure 4). 15% of our sequences coding for putative proteins did not show homology with the contig set. This result could be due to differences in the composition of the microbiota between the individuals sampled in each study. Although unlikely due to the high numbers of reads that contained the Qin et al. data set, it could be also that microbiota members that are present in low number and that would not be detected in the metagenomic study express certain genes at high levels [17]. Surprisingly, a 53% of the total non ribosomal sequences remained uncharacterized. When we relaxed the parameter of homology search, we obtained a reduction of the above fractions, 7% for protein coding cDNAs and 46% for uncharacterized cDNAs not present in the contig set. The large fraction of unassigned reads could correspond to novel RNA sequences such as unknown mRNAs, RNA regulatory elements or RNA viruses. In recent years, small RNAs (sRNA) have been described as untranslated regulatory elements that have key roles in important biological processes, such as amino acid and vitamin biosynthesis, virulence, stress response and quorum sensing [5], [62], [63], [64]. Recently, in an ocean water metatranscriptomic study Shi *et al.* [5], [35] have detected a large fraction of sRNAs. The authors related these sRNAs to the regulation of nutrient acquisition and energy metabolism in free-living planktonic bacterial communities. To investigate the representation of known sRNA families in our fraction of uncharacterized cDNAs, we searched the Rfam database [65]. We found that 18% of this fraction was assigned to sRNA families (Table S3 in SI). Of those, we mapped a small fraction (3%) of sRNAs in the contig set described by Qin et al. [17]. Additional studies should be done to explore the role of these regulatory elements in the gut microbial community and their relationship with health.

### Conclusions

Our RNA-based results indicated that the phylogenetic composition of the active intestinal microbiota is fairly uniform among individuals, in contrast to the larger differences observed with metagenomic data, and that this homogeneity further increases at the functional level. Our data may suggest a health related functional profile showing some differences with those indicated by the potential functions of predicted genes in DNA-based surveys. Additionally, we found that the distribution of bacterial families across functional categories is also rather homogenous. These results must be interpreted with caution because the sample size is not too large. However, this work provides a framework for further studies analyzing the relationship between active microbiota and health status and comparing gut microbiota composition in different physiologic conditions. Finally, this is the first report of the presence of small RNAs in the gut microbial community. Due to the important regulatory roles of these elements in prokaryotic physiology and pathogenicity, further research is needed to provide a deeper knowledge of their relationship with health and disease.

## Materials and Methods

### Sample collection

The ten healthy volunteers involved in this study provided their informed consent (Table S1 in SI). None had intestinal organic disorders or recent treatment with antibiotics. Fecal samples were collected in sterile containers containing 10 mL of phosphate buffered saline (PBS) (containing, per liter, 8 g of NaCl, 0.2 g of KCl, 1.44 g of Na2HPO4, and 0.24 g of KH2PO4 [pH 7.2]) and stored in home freezers until brought to the laboratory where samples were stored at −80°C until further processing.

### Total RNA isolation, mRNA amplification and cDNA synthesis

Total RNA was extracted using RiboPure-Bacteria kit (Ambion). mRNA was linearly amplified using the MessageAmp II-Bacteria kit (Ambion) according to the manufacturer's instructions. Briefly, since bacterial mRNAs have not a stable poly(A) tail the total RNA is polyadenylated using *Escherichia coli* poly(A) polymerase which facilitates preferential isolation of mRNAs from rRNAs. The next steps consisted in an in vitro transcription mediated linear amplification to increase the number of all mRNA molecules. The RNA was converted to double-stranded cDNA with random hexamers. Finally, 5 ug of cDNA were digested with *Bpm* I, purified, and used for pyrosequencing. The quantity and quality of the total RNA, antisense RNA and cDNA were assessed using the Nanodrop-1000 Spectophotometer (Thermo Scientific, Wilmington, DE) and standard agarose gel electrophoresis.

### Pyrosequencing

The cDNAs of each sample were sequenced by Life Sequencing (Valencia, Spain) with a Roche GS FLX sequencer and Titanium chemistry. The samples were pooled in two groups and sequenced on half a plate each.

### rRNA databases and SSU and LSU rRNA transcript identification

We used the Small Subunit rRNA Reference Database (SSUrdb) and Large Subunit rRNA Reference Database (LSUrdb) described in Urich *et al.* [34]. In order to select the correct parameters for the BLASTN comparisons, we used SSU, LSU and mRNA test sets. 1000 SSU human gut associated sequences were collected from the environmental division of the NCBI through the envDB database [66]. The same number of LSU and mRNA sequences was collected from Genbank using regular expressions to minimize contamination. Fragments of 100 bp were obtained by randomly sampling out the obtained sequences. We compared these datasets with the SSUrdb and LSUrdb using BLASTN with different maximum e-values. This analysis showed that an e-value threshold of 10^−16^ for the SSUrdb and 10^−4^ for LSUrdb give the lowest rates of “cross-contamination”.

All the sequences shorter than 60 bp were left out of the current analysis. The remaining cDNA sequences were compared to the SSUrdb described in Urich *et al.* [34] using BLASTN. All sequences with positive matches were labeled as 16S rRNAs and used to determine the phylogenetic diversity of the active bacteria. The remaining cDNAs were compared to the LSUrdb, all 23S putative sequences were discarded and the remaining fraction was used to determine the functional content of the sample.

### Phylogenetic analysis of 16S RNAs

The taxonomic information of the 16S rRNA transcripts was obtained by comparison against The Ribosomal Database Project-II (RDP) [46]. This method is widely used and provides rapid taxonomic classifications from domain to genus of both partial and full-length rRNA gene sequences. We considered only annotation with a bootstrap value over 0.5, stopping the assignation at the last well identified phylogenetic level and leaving successive levels as unclassified (uc).

### Functional analysis of putative mRNAs

All cDNA sequences with no significant homology with any of the rRNA databases (27,923 reads) were aligned to the NCBI-nr protein database (released 19 September 2009) using BLASTX [55]. Sequences with detected homology were assigned to functional proteins (14,680 sequences) and their phylogenetic binning assessed using the MEGAN software [56]. MEGAN is a well-recognized tool for phylogenetic classification applicable to metagenomic and metatranscriptomic data. It is based on BLAST results and the assignment to the NCBI taxonomy is performed using the lowest common ancestor (LCA) algorithm.

A COG (Cluster Orthologous Group) reference sequence database was constructed using the COG annotated proteins present in all the 1012 completely sequenced bacterial genomes at NCBI (as of December 2009) (gCOGdb). This database contained 2,329,270 sequences distributed in functional categories. The sequences previously identified as putative proteins were compared with gCOGdb using BLASTX (default parameters except setting the maximum e-value to 10^−3^). All sequences assigned to more than one different non-overlapping COG function were discarded. Rate ratios were calculated using (n_c_/n)/(N_c_/N), where n_c_ is the number of hits to a given category “c” in our samples, n is the total number of hits in all categories in our samples, N_c_ is the number of hits to that category in gCOGdb and N is the number of hits to all categories in gCOGdb.

### sRNA homology analysis

We searched the Rfam database (version 9.1) [65] with the INFERNAL tool (version 1.0.2) [67] to identify the known sRNA families in the uncharacterized fraction of our data. The homology between our data and the Qin et al. [17] contig set was obtained using BLAT with 80% of the possible maximum alignment and a minimum sequence identity of 90%. For this analysis, we previously assigned as putative protein coding genes those of our sequences with an e-value ≤10^−3^ in the BLASTX search against NCBI-nr.

### Statistical analysis

We computed rarefaction curves as well as the Chao1 and the abundance-based coverage estimators (ACE) of richness [52], [53] to assess the expected number of unseen species in the samples. We also computed the Shannon index of biodiversity [54] to measure the level of heterogeneity in the taxonomic composition of the active microbiota. We also carried out a correspondence analysis to explore patterns of variation in the composition of the active microbiota across samples. We used a Bayesian statistical model to analyse the association between bacterial families and functional groups. Further details on the statistical analyses used are reported in the Supplementary Methods S1 in SI.

### Data deposition

All the cDNA sequences will be deposited in NCBI Short Read Archive under accession number SRA012604.11.

## Supporting Information

Methods S1 This file contains supplementary analysis.
(DOC)

Table S1 General characteristics of healthy volunteers.
(DOC)

Table S2 Biodiversity and richness estimators. Shannon's index of biodiversity (Shannon), Chao1 richness estimator (Chao1) and associated standard error (SE Chao1), Abundance Coverage Estimator (ACE) and standard error (SE ACE).
(DOC)

Table S3 Distribution of the reads in the Rfam families related to prokaryote regulation (represented by more than 10 sequences)
(DOC)

Figure S1 Distribution of the main active families in each sample. The average value was calculated from the global community composition.
(DOC)

Figure S2 Rarefaction curves calculated for each sample.
(DOC)

Figure S3 Correspondence analysis of samples surveyed.
(DOC)

Figure S4 Relative abundance of main families observed in 16S transcripts and mRNAs using RDP and Megan (LCA) as assignment method.
(DOC)
