# Supplementary material for: Metatranscriptomic Approach to Analyze the Functional Human Gut Microbiota
Source: PLoS One. 2011 Mar 8;6(3):e17447. doi: 10.1371/journal.pone.0017447 (PMC3050895; doi:10.1371/journal.pone.0017447)
Supplement: Methods S1 — This file contains supplementary analysis. (DOC) [file pone.0017447.s001.doc]

**Methods SI**

**Statistical analysis**

We used a Bayesian hierarchical model to analyze potential associations between bacterial families and functional groups. Let Y_ij_ be the number of sequences from family j=1,…,m in functional group i=1,…,n. Let E_ij_ be the number of expected sequences in function i and taxon j according to the standard null hypothesis of independence between functions and taxa. This hypothesis is equivalent to say that all families are present in a similar proportion in all the functions. Our model tries to detect departures from this null hypothesis. The specification is as follows. We first assume that Y_ij_ ~ Poisson(λ_ij_ E_ij_), that is, we assume that the number of observed counts will follow a Poisson distribution with mean the expected counts (under independence) E_ij_ times a factor λ_ij_ that measures the over- or under-representation. Values of λ_ij_ above 1 would indicate an over-representation of taxa j in function i. Conversely, values below 1 would suggest under-representation. We then assume that log(λ_ij_)=α+θ_i_+γ_j_+ν_ij_. That is, the variation of the λ_ij_ is then decomposed (on the log scale) into an overall intercept α plus function- and taxa-specific main effects θ_i_ and γ_j_, respectively, plus an interaction ν_ij_. These interactions are to pick up departures from the null hypothesis of independence between functions and taxa.

In the Bayesian framework, all the model parameters are assigned prior distributions that represent prior belief or information, if available. This prior information is then combined with the information provided by the data (through the likelihood) via Bayes theorem to produce the posterior distribution of the model parameters, which is the output of the Bayesian inference. In complex models, the posterior distribution often does not have an analytically closed expression and one needs to resort to the so-called Markov chain Monte Carlo (MCMC) simulation techniques to obtain a sample from the posterior distribution for each model parameter. This posterior sample in later on summarized to extract the relevant information, point estimates, quantiles, credibility intervals, etc. We chose a flat normal prior distribution (i.e. a normal distribution with large variance) for the intercept. For the taxa and function main effects we chose θ_i_ ~ Normal (0, σ_θ_) and γ_j_ ~ Normal(0, σ_γ_), respectively. For the interactions ν_ij_, we chose a so-called “spike and slab” prior distribution ν_ij_ ~ pNormal (0, σ_1_) + (1-p) Normal (0, σ_2_), which is a mixture of two zero-mean normal distributions, one with a large standard deviation (say σ_1_) and the other with a small one. The latter is to pick up noise basically, whereas the former is to detect sizeable associations between taxa and functions. We also assigned the following prior distributions to the standard deviations of the random effects: σ_θ_, σ_γ_ ~ InverseGamma(0.01, 0.01); σ_1_ ~ Normal(0, 100) to allow for the standard deviation σ_1_ to be potentially large, and σ_2_ ~ Normal(0, 0.3) to ensure that it is small. The model was fitted using the license-free statistical package for Bayesian inference JAGS (http://www-fis.iarc.fr/~martyn/software/jags/).The statistical analyses were carried out with the free statistical package R (R Development Core Team 2009) and its libraries “vegan” (Oksanen et al. 2008) and “rjags” (Plummer 2009)".

**References**

Oksanen J, Kindt R, Legendre P, O'Hara B, Simpson GL, Solymos P, Stevens MHH, Wagner H. 2008. Vegan: Community ecology package. R package version 1.15-1. http://cran.r-project.org/, http://vegan.r-forge.r-project.org/.

Plummer M. 2009. rjags: Bayesian graphical models using MCMC. R package version 1.0.3-11. http://CRAN.R-project.org/package=rjags.

R Development Core Team. 2009. R: A language and environment for statistical computing. R Foundation for statistical computing, Vienna, Austria. ISBN 3-900051-07-0, URL http://www.R-project.org.
