# Supplementary material for: Metatranscriptomic Approach to Analyze the Functional Human Gut Microbiota
Source: PLoS One. 2011 Mar 8;6(3):e17447. doi: 10.1371/journal.pone.0017447 (PMC3050895; doi:10.1371/journal.pone.0017447)
Supplement: Table S1 — General characteristics of healthy volunteers. (DOC) [file pone.0017447.s002.doc]

Table S1: General characteristics of healthy volunteers

| Sample | Age | Sex | Nationality |
| --- | --- | --- | --- |
| A | 62 | F | Spain |
| B | 55 | M | Spain |
| C | 29 | F | Spain |
| D | 42 | F | Spain |
| E | 37 | M | Spain |
| F | 36 | M | Mexico* |
| K | 26 | F | Spain |
| L | 21 | M | Spain |
| N | 28 | M | Spain |
| O | 57 | F | Spain |

*Volunteer (sample F) was 8 months in Spain
