# Supplementary material for: Metatranscriptomic Approach to Analyze the Functional Human Gut Microbiota
Source: PLoS One. 2011 Mar 8;6(3):e17447. doi: 10.1371/journal.pone.0017447 (PMC3050895; doi:10.1371/journal.pone.0017447)
Supplement: Table S2 — Biodiversity and richness estimators. Shannon's index of biodiversity (Shannon), Chao1 richness estimator (Chao1) and associated standard error (SE Chao1), Abundance Coverage Estimator (ACE) and standard error (SE ACE). (DOC) [file pone.0017447.s003.doc]

Table S2: Biodiversity and richness estimators. Shannon’s index of biodiversity (Shannon), Chao1 richness estimator (Chao1) and associated standard error (SE Chao1), Abundance Coverage Estimator (ACE) and standard error (SE ACE)

| Sample | N | Shannon | Chao1 | SE.Chao1 | ACE | SE.ACE |
| --- | --- | --- | --- | --- | --- | --- |
| A | 48 | 2.23 | 48.38 | 1.12 | 49.69 | 3.45 |
| B | 33 | 1.50 | 34.43 | 2.51 | 37.30 | 3.00 |
| C | 41 | 2.21 | 42.43 | 2.51 | 43.90 | 3.24 |
| D | 39 | 2.47 | 40.5 | 7.19 | 40.44 | 2.95 |
| E | 31 | 1.95 | 40.33 | 16.49 | 38.49 | 2.81 |
| F | 28 | 2.22 | 29.43 | 2.51 | 33.89 | 3.08 |
| K | 40 | 2.34 | 42.14 | 3.24 | 44.80 | 3.22 |
| L | 45 | 2.14 | 50.14 | 5.88 | 52.70 | 3.52 |
| N | 37 | 2.44 | 37.60 | 1.77 | 38.87 | 3.11 |
| O | 46 | 2.33 | 64.20 | 18.6 | 76.70 | 4.19 |

N: total number of observed families
