# Supplementary material for: Metatranscriptomic Approach to Analyze the Functional Human Gut Microbiota
Source: PLoS One. 2011 Mar 8;6(3):e17447. doi: 10.1371/journal.pone.0017447 (PMC3050895; doi:10.1371/journal.pone.0017447)
Supplement: Table S3 — Distribution of the reads in the Rfam families related to prokaryote regulation (represented by more than 10 sequences) (DOC) [file pone.0017447.s004.doc]

Table S3: Distribution of the reads in the Rfam families related to prokaryote regulation (represented by more than 10 sequences)

| Rfam id | Number of reads | Annotation | Function |
| --- | --- | --- | --- |
| RF00035 | 11 | OxyS | Gene regulation |
| RF01338 | 11 | CRISPR-DR25 | Prokaryotic immune system |
| RF00518 | 12 | speF | Polyamide biosynthesis |
| RF00627 | 12 | P15 | 3-deoxy-7-phosphoheptulonate synthase gene expression |
| RF01057 | 12 | SAH_riboswitch | S-adenosylhomocysteine biosynthesis regulation |
| RF01372 | 12 | CRISPR-DR29 | Prokaryotic immune system |
| RF00021 | 13 | Spot_42 | Galactose operon regulation |
| RF00534 | 13 | SgrS | Glucose-phosphate stress |
| RF00521 | 14 | SAM_alpha | Riboswitch: S-adenosyl methionine/methionine biosynthesis |
| RF01066 | 14 | 6C | Gene regulation during sporulation |
| RF01402 | 14 | STnc150 | Hfq binding |
| RF01406 | 14 | STnc500 | Hfq binding |
| RF00011 | 15 | RNaseP_bact_b | Ribozyme: Maturation of tRNA |
| RF01411 | 15 | BsrF | Gene regulation in vegetative growth phase |
| RF01412 | 15 | BsrG | Gene regulation in vegetative growth phase |
| RF00013 | 18 | 6S | Gene regulation in stationary growth phase |
| RF01328 | 18 | CRISPR-DR17 | Prokaryotic immune system |
| RF01394 | 19 | isrK | Hfq binding |
| RF00014 | 20 | DsrA | Stress sigma factor, RpoS. |
| RF00110 | 20 | RybB | Envelope stress response |
| RF00234 | 20 | glmS | Glucosamine-6-phosphate synthesis |
| RF00435 | 21 | ROSE | Heat shock gene expression |
| RF00230 | 22 | T-box | Translation associated genes |
| RF01352 | 22 | CRISPR-DR43 | Prokaryotic immune system |
| RF00128 | 23 | GlmY_tke1 | Glutamine synthase regulation |
| RF01386 | 23 | isrB | Hfq binding |
| RF01400 | 23 | istR | Hfq binding |
| RF00122 | 25 | GadY | Acid resistance gene regulation |
| RF01397 | 25 | isrO | Hfq binding |
| RF01056 | 26 | Mg_sensor | Magnesium transporter protein regulation |
| RF00166 | 28 | PrrB_RsmZ | Secondary metabolism regulation |
| RF00050 | 29 | FMN | Riboswitch: flavin mononucleotide biosynthesis |
| RF00463 | 31 | ApoB_5_CRE | ApoB regulation |
| RF00040 | 32 | rne5 | RNAse E regulation |
| RF01384 | 33 | InvR | Outer cell membrane porin protein regulation |
| RF00018 | 34 | CsrB | Carbohydrate metabolism regulation |
| RF00083 | 35 | GlmZ_SraJ | Glutamine synthase regulation |
| RF00169 | 41 | SRP_bact | Traffic of proteins |
| RF01395 | 44 | isrL | Hfq binding |
| RF01360 | 46 | CRISPR-DR47 | Prokaryotic immune system |
| RF00506 | 47 | Thr_leader | Thr operon regulation |
| RF01408 | 47 | sraL | Hfq binding |
| RF01053 | 48 | Deinococcus_Y_RNA | Ro ribonucleoprotein particle regulation |
| RF00514 | 49 | His_leader | His operon regulation |
| RF00023 | 50 | tmRNA | Rescue stalled ribosomes |
| RF00005 | 51 | tRNA | Protein synthesis |
| RF00001 | 55 | 5S_rRNA | Ribosome assembly |
| RF00373 | 57 | RNaseP_arch | Ribozyme: Generation of mature tRNA |
| RF00552 | 60 | rncO | rnc regulation |
| RF00174 | 62 | Cobalamin | Cobalamin riboswitch |
| RF01402 | 63 | STnc490k | Hfq binding |
| RF01372 | 64 | CRISPR-DR59 | Prokaryotic immune system |
| RF00084 | 67 | CsrC | Carbohydrate metabolism regulation |
| RF00512 | 76 | Leu_leader | Leucine biosynthetic operon regulation |
| RF01065 | 80 | 23S-methyl | rRNA methyltransferases regulation in Lactobacillales order |
| RF00632 | 100 | sxy | Competence regulation |
| RF01071 | 110 | OLE RNA | Ribozyme in Clostridiales order |
| RF01390 | 129 | isrG | Hfq binding |
| RF00010 | 167 | RNaseP_bact_a | Ribozyme:Maturation of tRNA |
