# Supplementary material for: Metatranscriptomic Approach to Analyze the Functional Human Gut Microbiota
Source: PLoS One. 2011 Mar 8;6(3):e17447. doi: 10.1371/journal.pone.0017447 (PMC3050895; doi:10.1371/journal.pone.0017447)
Supplement: Figure S2 — Rarefaction curves calculated for each sample. (DOC) [file pone.0017447.s006.doc]

Fig. S2. Rarefaction curves calculated for each sample.
